# A cross sectional study of growth of children with sickle cell disease, aged 2 to 5 years in Yaoundé, Cameroon

**DOI:** 10.11604/pamj.2019.34.85.16432

**Published:** 2019-10-13

**Authors:** Suzanne Sap Ngo Um, Judith Seungue, Anastasie Yanda Alima, Ritha Mbono, Hubert Mbassi, David Chelo, Paul Olivier Koki

**Affiliations:** 1 Mother and Child Center of Yaoundé, Yaoundé, Cameroon; 2 Faculty of Medicine and Biomedical Sciences of the University of Yaoundé I, Yaoundé, Cameroon

**Keywords:** Sickle cell disease, growth, WHO norms, sub-Saharan Africa

## Abstract

**Introduction:**

Growth of children affected by Sickle Cell Disease (SCD) is not well described in sub-Saharan Africa despite the high prevalence of the disease. Few data are available in this context and on the issue using the World Health Organization growth norms. We therefore conduct the present study with the aim of describing the growth of affected children aged less than 5 years. We also assessed correlation of anthropometric parameters with disease severity criteria.

**Methods:**

A cross-sectional study was conducted during a period of 8 months, at the Mother and Child Center of Yaoundé. The sample included 77 children with SCD aged 2 to 5 years old in steady state. Anthropometric measurements and socio-demographic data were collected and analyzed. All statistical tests were two-tailed with p<0.05 considered significant.

**Results:**

Median age of study population was 3.67 years. Low weight, height and weight for height Z-scores (<-2SD) were observed in 4%, 4%, and 5% of children, respectively. Projection of these parameters were stackable on WHO curves. Regression analysis indicated an association of low height-for-age and of low Body Mass Index (BMI)-for-age with age.

**Conclusion:**

This study demonstrates unexpectedly lower mean Z-score for weight, height and weight for height than reported while using WHO norms.

## Introduction

Sickle Cell Disease (SCD) is the most prevalent genetic disease in the world with a clear predominance in the black population [1, 2]. This hemoglobinopathy is a major public health problem in Africa [1-5] and particularly in Cameroon, where about 20 to 25% of the population carries the sickle cell trait [1, 6]. Numerous acute and chronic complications are responsible for high morbidity and mortality in affected patients [1, 7]. Chronic complications include stunting and delayed puberty, particularly in homozygous patients, mainly because of increased basal metabolism related to hemolysis and chronic inflammation, endocrine disorders related to free iron toxicity on endocrine organs [7], multiple morbid episodes, micronutrient deficiency, [2, 8-14], and probably low socio-economic level [8, 14]. Although growth pattern in SCD has been studied, few African data are available, particularly in Cameroon [1, 15]. Most of the studies on growth used ethnical or specific growth charts [5, 9, 11-12]. We hypothesized that growth of children less than five, affected by sickle cell anemia, in a tertiary care center, may be near normal using WHO norms [16-18]. We therefore proposed to describe the anthropometric parameters of a population of children aged 2 to 5 years with homozygous sickle cell disease (Hb SS), according to the growth standards of the World Health Organization (WHO), then to identify the relationship between these parameters and severity criteria of the disease (clinical and hematological in a single center).

## Methods

**Type of study:** we conducted a cross sectional study (January to August 2014) in a single referral centre-the Chantal Biya Foundation's Mother and Child Center. Consecutive sampling included all children with homozygous SCD who came for routine consultation and whose parents or guardians gave their informed consent. We included patients aged 2 to 5 years in steady state. We excluded all those with another documented chronic condition that may interfere with growth (chronic kidney disease, heart disease) or alter the measurement of the size (spinal abnormalities, inequality of members). The sample size was estimated according to the Cochran formula: N = ((Z√ (p (1-p))) / δ)², giving a minimum size of 73 patients. (N=sample size, p=prevalence, δ=precision, Z=standardized significance level at 1.96 for a 95% confidence interval). We considered a prevalence of 5% [1, 2].

Socio-demographic data and co-morbidity factors related to the severity of the disease were collected by the interview and the patient follow-up record. The definition of clinical events was made according to that proposed by the Co-operative Study of Sickle Cell Disease (CSSD) [15]. Height and weight were measured to the nearest 0.1cm and 0.05kg using a Leicester Height Measure^®^ MK II stadiometer and a Tanita^®^ BC-351 scale respectively. Body Mass Index (BMI) was calculated by dividing weight (in kg) by the square of height (in meter): W/H². These variables were projected on WHO standards and expressed in Z-scores. Stunting, underweight, and wasting were defined by Z-score <-2 for height-for-age (HAZ), weight-for-age (WAZ) and BMI-for-age (BMI-Z), respectively. Stunting was considered mild for a Z-score between -1 and -2; moderate for a Z-score between -2 and -3 and severe for a Z-score <-3. Overweight and obesity were defined by IMC-Z respectively above +2 and above +3 Z-score. From the hematological point of view, the following tests were done (in steady state): hemolysis factors: Hemoglobin (Hb), Lactate Dehydrogenase (LDH), free bilirubin; medullary activity factors: Mean Corpuscular Volume (MCV), reticulocyte, platelet and Fetal Hb (HbF) levels.

The hemogram was performed by fluorescence and flow cytometry on a PentraDX Nexus^®^ PLC. The number of reticulocytes were counted according to a manual technique after staining with brilliant blue cresyl or una blue. A kinetic type of spectrophotometry was used to determine LDH level and a colorimetric method for free bilirubinemia on a Vitros^®^ automaton for dry chemistry. We used hemoglobin electrophoresis at alkaline PH (on cellulose acetate) to determine the type of SCD.

**Statistical analysis:** after a preliminary phase of descriptive analysis (estimation of means, medians, frequencies), the correlation between the independent variables (age, sex, factors related to the severity of the disease or factors of co-morbidity), and the dependent variables (growth, weight, height and BMI for age) indicators were searched for by univariate analysis and logistic regression according to Pearson correlation for continuous variables and Anova for categorical variables. During the univariate analysis, we considered a p value significant <0.2. To control confounding factors and interactions between explanatory variables, we performed a multivariate analysis to highlight the only factors influencing growth of our study population. The binary logistic regression allowed to highlight the relative risk factors (Odds Ratio) and the correlation was significant for a p value <0.05.

**Ethical considerations:** we received ethical approval from the Institutional Committee of Ethics and Research of the Faculty of Medicine and Biomedical Sciences of the University of Yaoundé I. A written consent was obtained from the parents or guardians and the data remained confidential according to Helsinki declaration.

## Results

**General characteristics of the study population:** we included 77 patients from which 36 were girls. The median age of patients was 3.67 years old. The diagnosis of Myelodysplastic Syndromes (MDS) was made at a median age of 14 months. More than half of the patients n=45 (58.44%) had already been transfused at least once. (Table 1) There was at least one hospitalization for severe vaso-occlusive crisis in 69 (89.6%) of them. Hematologic parameters of bone marrow activity and haemolysis were similar in girls and boys with the exception of leukocyte count, which was higher in boys (p=0.03) (Table 2).

**Table 1:** General characteristics of the study population

Variables	
Sex F/M	36/41
Age in years Median (IQR)	3.67 (2.6 – 4.71)
Age at diagnosis in months Median (IQR)	14 (8-29)
Type of SCD	SS: 76 (98.7)
n (%)	Sβ: 1 (1.3)
Number of VOC/year Median (IQR)	2 (1-4)
Number of previous transfusions (IQR)	1 (0- 3.5)
Folic Acid: Yes/No (%)	69 (90)/ 8(10)
Hydroxyurea : Yes/No (%)	8 (10)/69 (90)
Hydratation Normal/Insufficient (%)	46 (59.7)/ 31 (40.2)
Antibioprophylaxy Yes/No (%)	60 (84.5)/17 (15.5)

**Table 2:** Haematological profile of the study population

Variables*	Girls	Boys	p
Hb, g/dl	7.6 (7-8.1)	7.2 (6.2 -8.1)	0.21
MCV, fl	80.40 (75-92)	82 (75-89)	0.9
Leucocytes (x10^3^ mm^3^)	13.50 (11.50-16.60)	16.69 (13.40-18.54)	0.03
Platelets (x10^3^ /mm^3^)	379.50 (287.50-522.50)	337 (300 – 469)	0.3
Reticulocytes (x10^3^ /mm^3^)	282 (179.26-315.74)	228.8 (196.04 – 360.21)	0.57
LDH (UI /L)	1289 (1176 -1567)	1681 (1179 – 1986.50)	0.36
Bilirubin (mg/L)	20.70 (13.40-41)	28.45 (14.85-34.75)	0.9
Hb F (%)	22.80 (14.90 – 32.20)	21.45 (14.50-25.25)	0.69

* variables are expressed in mean (SD)

**Figure 1:**
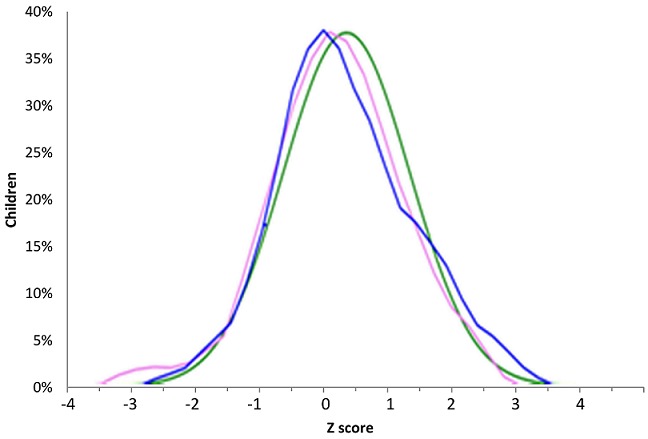
Projection of the WAZ of children on WHO norms (green: WHO norms, pink: girls (n=36), blue: boys (n=41)

**Figure 2:**
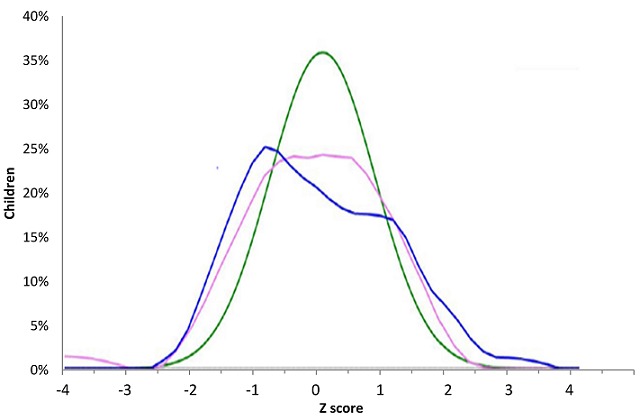
Projection of Height (HAZ) on WHO norms. (green: WHO norms, pink: girls (n=36), blue: boys (n=41))

**Figure 3:**
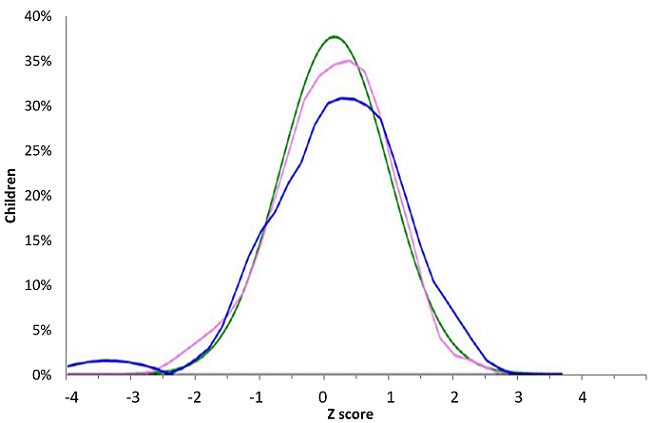
Projection of BMI on WHO norms. (green: WHO norms, pink: girls (n=36), blue: boys (n=41)

**Anthropometric parameters:** we found underweight in 3 (4%) patients, WAZ between -2 and -1 in 14 children (18%) and normal WAZ in 59 patients. WAZ projection on WHO curves was similar (Figure 1, Table 3). Stunting was found in 3 (4%) patients, emaciation in 22 (29%) and normal height in 49 patients (63.6%). The median size of the study population was -0.53 Z-score (-1.30, 0.57) (Table 3). Projection of HAZ on WHO curves showed for boys 2 peaks reflecting a tendency to growth retardation. Amplitude for both sexes was smaller than the WHO one (Figure 2). Wasting was found in 4 children (5%), WHZ was between -2 and -1 Z-score in 16 children (21%) and in normal value in 55 (71.4%) children of our study population. The median BMI (interquartile range (IQR)) was -0.38 (-1.07, 0.43) and the projection was stackable on WHO curve (Figure 3).

**Table 3:** Mean Z-score of growth parameters of the study population

		Height for age		Weight for-age		BMI for age	
Variables	n	Z-score	SD	Z-score	SD	Z-score	SD
All	77	-0.15	1.32	-0.09	1.00	-0.01	1.09
Boys	41	-0.07	1.31	-0.02	1.02	0.02	1.20
Girls	36	-0.23	1.33	-0.17	0.99	-0.04	0.97
**Age (years)**							
[2-3]	31	-0.35	1.47	-0.14	1.15	0.11	1.19
[3-4]	17	-0.30	1.32	-0.24	0.89	-0.08	1.08
[4-5]	29	0.16	1.11	0.05	0.89	-0.08	1.00

**Relationship with the severity criteria:** the severity score of the disease was low in our study population. For a threshold of significance at 5%, no correlation was found between the severity criteria of the pathology and the growth indicators. Nevertheless, when this threshold was set at 20% as defined by Pearson, it was noted that the older the child, the more likely he was to have a stunted delay, wasting or underweight. This risk of delayed stature also increased with the number of hospitalizations for severe CVO. The BMI seemed to be influenced by the increase in the number of transfusions, and the LDH levels. There were conflicting results regarding the correlation between platelet levels, reticulocyte levels and PAZ, between leukocyte levels and BMI, respectively (Table 4).

**Table 4:** Correlation between severity criteria and growth parameters (univariate analysis)

Correlation (p-value)	WAZ	HAZ	BMIZ
Age	-0,255 (0,002)*	-0,226 (0,002)*	-0,389 (0,000)*
VOC (number of hospitalisation)	-0,077 (0,369)	-0,099 (0,184)*	-0,056 (0,448)
Transfusion	-0,093 (0,418)	-0,110 (0,246)	-0,157 (0,096)*
Hb level (g/dl)	0,0978 (0,378)	0,0732 (0,446)	-0,025 (0,789)
Hb F (%)	0,0354 (0,719)	0,0589 (0,507)	0,1063 (0,230)
MCV	-0,114 (0,307)	-0,115 (0,236)	-0,006 (0,947)
Leucocytes count	0,0781 (0,482)	-0,070 (0,470)	0,243 (0,011)*
Platelets count	0,1838 (0,102)*	0,0116 (0,906)	0,0920 (0,350)
Reticulocytes count	0,1904 (0,141)*	0,0627 (0,580)	0,221 (0,048)*
LDH	-0,031 (0,801)	0,1059 (0,331)	-0,177 (0,101)*
Bilirubin	0,0589 (0,660)	0,0623 (0,597)	0,0014 (0,990)

* Pearson correlation significant when p value considered at 20%. Only variables with (*) were included in multivariate analysis

VOC vaso occlusive crisis, Mean corpuscular volume, LDH lactico dehydrogenase

## Discussion

We evaluated the growth of 77 children aged 2 to 5 years with MDS. Our goals were to describe height, weight, BMI, in light of WHO standards; and finally to look for a possible correlation between the manifestations of severity of the disease and these growth indicators. Our study was cross sectional, limiting analysis to growth velocity of each patient. In addition, patients enrollment was done in a specialized service, thus the results cannot be extrapolated to rural areas. Nevertheless, the results suggest that despite the difficult conditions of care in developing countries, children with sickle cell disease can have a similar growth to non-affected children.

**General characteristics of the study population:** the median age of our study was lower than that found by other authors because we chose to study exclusively under 5 years. This choice is due to a concern for the homogeneity of the study population and the desire to use WHO standards [16-20]. Our population was similar to that of Silva *et al.* who studied children aged 5 months to 8 years [11]. The median age at diagnosis was 14 months close to that of Al Saqladi *et al.* (1 year) [9]. This age also differed from studies in western countries where neonatal screening is performed [10, 13, 14]. Newborn screening is not yet routinely done in our setting and diagnosis is evoked at the beginning of clinical manifestations. Disease was less severe in our study population probably due to young age as the trend of severity increase with age [13, 15]. Moreover, children were followed in a specialized care unit and this can also explain the few signs of severity observed.

**Hematological profile of the study population:** the SS genotype was most represented in our study population. The other authors had a larger proportion of Sβ subjects [21, 22]. This difference could be explained by the fact that, genotypic diagnostic techniques are different. Indeed, the diagnosis of homozygous SCD in our study population was determined by HB electrophoresis at alkaline pH. This technique does not allow accurate quantification of Hb A2. The proportion of Sß could therefore be underestimated in our population. A high proportion of children had a high Hb F (>15%) with a median rate of 22%, above those of Al-Saqladi *et al.* (4.4%) [8, 12]. This difference in Hb F level could be explained by type of haplotypes [21]. In fact, there is a correlation between haplotype and hemoglobin F levels in one hand, haplotype and hemoglobin level on the other hand. The Benin haplotype, most found in our setting, is associated with a higher level of Hb F hemoglobin [21, 22]. The average hemoglobin level was 7.2g/dl, as in the under-5 population of Al-Saqladi *et al.* [12]. The mean free bilirubin level was approximately 2-fold higher than normal, as in the study populations of Al-Saqladi *et al.* In the study population of the latter, this rate increased significantly with age (p=0.005) [12]. The average levels of reticulocytes (241060/mm^3^) and leucocytes (14525/mm^3^) are about 1.5 and 2 times higher than the upper limits of normal values. The Al-Saqladi *et al.* study found similar levels for reticulocytes but lower for leucocytes [12].

**Anthropometric parameters of the study population:** emaciation was found in 4% of the study population. This result is similar to that of Silva *et al.* [11] in Brazil and similar to the rate in general urban population (3.4%) of the same age [19]. Stunting was found in 4% of patients. This is greater than results from Silva *et al.* (1.4%) and Patey *et al.* [11, 20]. Optimal management performed in developed countries may explain this difference [16, 20, 23] and also the type of study (case control in the Patey *et al.* study). Wasting affected 5% of our study population. These results are largely lower than those found in other countries of central Africa [24-27], but higher than the one in general urban area in our country (2.4%) [19]. Differences amongst studies may be also related to types of norms used for comparison. The Patey *et al.* study suggested the use of appropriate norms or growth curves on ethnical point of view as growth pattern may differ in-between specific ethnic groups [20]; but the issue of ethnicity has been already addressed by WHO as children from all continents were included to realize actual norms. Therefore these norms are accurate for normal general population [17, 19]. Interestingly, projection of anthropometric parameters of our study population was superimposed to WHO standards. This may suggest that growth pattern of SCD patients, under five years, with appropriate care, is slightly similar to normal population. The question could be raised for older children.

This almost adequate growth of children under 5 years of age may be related to high levels of Hb F, haplotype differences, or low bone marrow activity. Indeed, an association exists between the increase of the rate of HBF and the improvement of the growth on the one hand; the increase of the HbF rate and the reduction of morbid events, therefore of energy expenditure, on the other hand [23, 27, 28]. In addition, the Hb F level and the hemoglobin level could be correlated with the type of haplotype [21, 22, 28]. Benin haplotye is associated with moderate forms of sickle cell disease expression and higher levels of HbF and Hb in carriers [21, 22, 28]. This is different for the Bantu haplotype and the Arab-Indian haplotype. The Caruso-Nicoletti *et al.* study found in his cohort a predominant benign haplotype, partially explaining the low weight deficit found [28]. The Mukherjee *et al.* study on the other hand, found significant growth retardation in his study population, carrying mostly Indian haplotype [29]. The mean LDH is not very elevated, which indicates that hemolysis is low. The low level of reticulocyte in our population, is suggestive of little erythropoietic bone marrow activity. This may also explain adequate growth in our study population. The relationship between proteins turnover (in this case glutamine) and the increase in rest energy expenditure has been shown [30]. Indeed, glutamine, the most abundant amino acid in humans, whose endogenous production is reduced with age, is the “fuel” of choice for fast-growing cells such as reticulocytes. Salman *et al.* have shown that its use in sickle cell patients is 47% higher than in normal subjects and is associated with a 19% increase in resting energy expenditure and 60% in cardiac output. These variations can be attributed to the increase in Hb synthesis and cardiac work. [31]. Thus, the Williams *et al.* study showed that administration of oral glutamine to sickle cell patients reduces energy expenditure by 6%, improving their BMI and body fat composition [31]. Based on these evidences, our study population has low energy expenditure and probably endogenous synthesis of glutamine enough to compensate resting energy expenditure and improve growth.

## Conclusion

The present work shows that 5% of the population of sickle cell children studied have growth impairment. Anthropometric parameters are stackable on WHO norms. Therefore, WHO growth standards may be appropriate for sickle cell patients aged less than five.

### What is known about this topic

- Growth of children affected by sickle cell is severely impaired;
- There is a need of special growth curves for them.

### What this study adds

- Growth of children less than 5 years is comparable to WHO norms in tertiary care center in Cameroon;
- There is no need for special growth curves for children less than 5 years affected by sickle cell; the WHO norms may be appropriate for their growth assessment.

## Competing interests

The authors declare no competing interests.
